# 1623. Assessing Clinical Evaluation and Patterns of Treatment in Nationally Reported Cases of Congenital Syphilis in Liveborn Infants – United States, 2016–2021

**DOI:** 10.1093/ofid/ofad500.1458

**Published:** 2023-11-27

**Authors:** Kevin P O’Callaghan, Michelle L Johnson Jones, David A Jackson, Elizabeth Torrone

**Affiliations:** Centers for Disease Control and Prevention, Powder Springs, Georgia; Centers for Disease Control & Prevention, Atlanta, Georgia; Centers for Disease Control and Prevention, Powder Springs, Georgia; Centers for Disease Control and Prevention, Powder Springs, Georgia

## Abstract

**Background:**

Congenital syphilis (CS) rates have increased in the United States since 2013. Management of infants exposed to syphilis during pregnancy entails a detailed clinical evaluation, followed by stratified work-up and treatment.

**Methods:**

During 2016─2021, 9,827 surveillance cases of CS were notified to the National Notifiable Diseases Surveillance System. Using the clinical framework described in the 2015 CDC Sexually Transmitted Diseases Treatment Guidelines, we assigned a clinical scenario (Proven or Highly Probable CS, Possible CS, CS Less Likely, and CS Unlikely) to 8,280 liveborn cases which had sufficient data for case classification (Figure 1). Based on data documented in the case report, we described the elements of evaluation and therapy provided to neonates, assessing whether they met, were in excess of, or were less extensive than recommendations, and stratified these findings by race, ethnicity, and geography.

**Figure:**
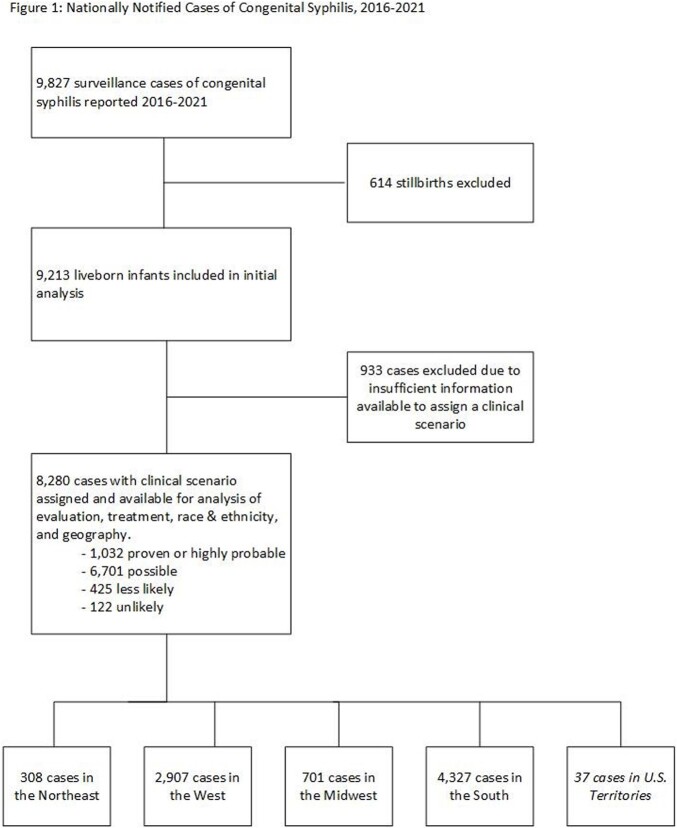

**Results:**

Of the 8,280 surveillance cases which were assigned a clinical scenario, the majority (80.9%) were classified as Possible CS, of whom 60.7% received an appropriate evaluation, while 68.4% received appropriate treatment. Of those assigned to Proven or Highly Probable CS, 46.0% received an appropriate evaluation, while 82.3% received appropriate treatment.

By U.S. census region, infants born in the Northeast were most likely to receive evaluation (75.7%) and treatment (87.3%) at or above recommendations for their case scenario, while infants born in the South were least likely to receive at or above recommended evaluation (49.5%) and treatment (59.3%). Infants born to a birth parent reporting non-Hispanic Native Hawaiian/Pacific Islander race were most likely to report less than recommended evaluation (36.7%), while infants of a non-Hispanic Black birth parent were most likely to experience less than recommended treatment (32.2%).

**Figure:**
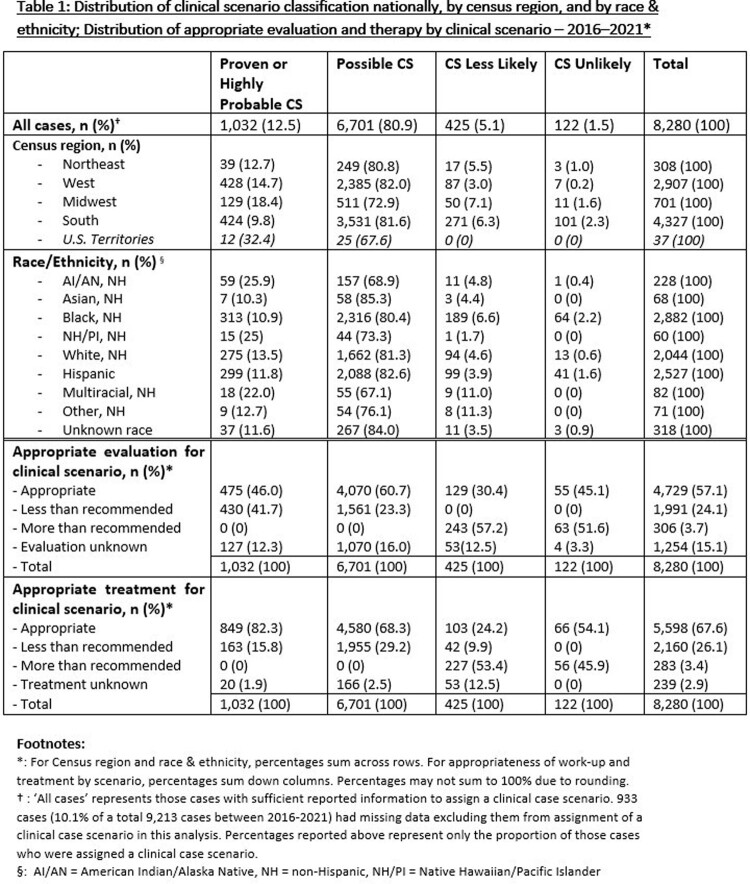

**Figure:**
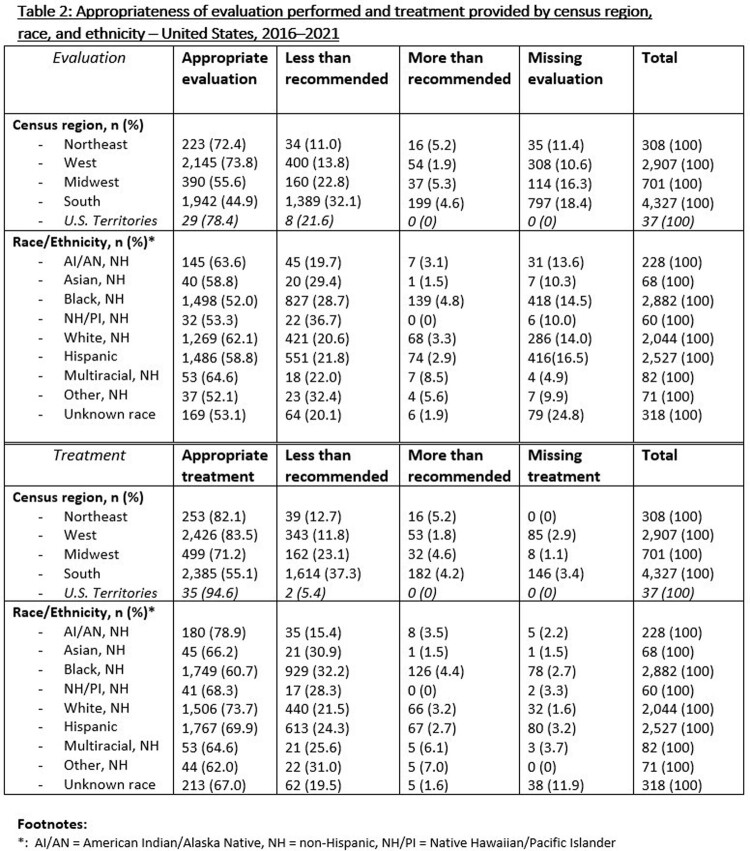

**Conclusion:**

This analysis of CS clinical scenarios underscores disparities in evaluation and therapy across geographic, racial, and ethnic lines. Further research is needed to understand factors involved in the evaluation and treatment of infants affected by CS, particularly for those born in the South and to birthing parents reporting minority racial and ethnic backgrounds.

**Disclosures:**

**All Authors**: No reported disclosures

